# The Egocentric Nature of Action-Sound Associations

**DOI:** 10.3389/fpsyg.2016.00231

**Published:** 2016-02-23

**Authors:** Nicole Navolio, Guillaume Lemaitre, Alain Forget, Laurie M. Heller

**Affiliations:** ^1^Auditory Perception Lab, Department of Psychology, Carnegie Mellon University, PittsburghPA, USA; ^2^Department of Human-Computer Interaction, Carnegie Mellon University, PittsburghPA, USA; ^3^CyLab Usable Privacy and Security Research Group, Carnegie Mellon University, PittsburghPA, USA

**Keywords:** auditory perception, environmental sounds, gestures, priming, egocentric, exocentric, frame of reference

## Abstract

Actions that produce sounds infuse our daily lives. Some of these sounds are a natural consequence of physical interactions (such as a clang resulting from dropping a pan), but others are artificially designed (such as a beep resulting from a keypress). Although the relationship between actions and sounds has previously been examined, the frame of reference of these associations is still unknown, despite it being a fundamental property of a psychological representation. For example, when an association is created between a keypress and a tone, it is unclear whether the frame of reference is egocentric (*gesture*-sound association) or exocentric (*key*-sound association). This question is especially important for artificially created associations, which occur in technology that pairs sounds with actions, such as gestural interfaces, virtual or augmented reality, and simple buttons that produce tones. The frame of reference could directly influence the learnability, the ease of use, the extent of immersion, and many other factors of the interaction. To explore whether action-sound associations are egocentric or exocentric, an experiment was implemented using a computer keyboard’s number pad wherein moving a finger from one key to another produced a sound, thus creating an action-sound association. Half of the participants received egocentric instructions to move their finger with a particular *gesture*. The other half of the participants received exocentric instructions to move their finger to a particular *number* on the keypad. All participants were performing the same actions, and only the *framing* of the action varied between conditions by altering task instructions. Participants in the egocentric condition learned the gesture-sound association, as revealed by a priming paradigm. However, the exocentric condition showed no priming effects. This finding suggests that action-sound associations are egocentric in nature. A second part of the same session further confirmed the egocentric nature of these associations by showing no change in the priming effect after moving to a different starting location. Our findings are consistent with an egocentric representation of action-sound associations, which could have implications for applications that utilize these associations.

## Introduction

It has been well established that environmental sounds portray information about our surroundings, such as event properties (Ballas, 1993; Houix et al., 2012) or as symbolic icons for nouns and verbs (Keller and Stevens, 2004; Giordano et al., 2010). Although it is clear that objects and actions can be represented by their accompanying sounds, it seems that *action*, rather than the *object* is most important in sound event perception. When asked to identify environmental sounds in a free identification task, people generally describe the actions that generated the sounds (Vanderveer, 1979). Additionally, a recent study found that listeners are better at identifying the action that caused a sound than they are identifying the object properties, such as material (Lemaitre and Heller, 2012). In fact, Lemaitre and Heller (2012) found that listeners were faster at identifying the action of a sound, even for a selection of sounds in which the actions and materials were equally identifiable. Neuroimaging studies also suggest that there are interactions between actions and sound processing in that action sounds activate more motor and pre-motor areas compared to control sounds (e.g., meaningless noise) (Aziz-Zadeh et al., 2004; Lewis et al., 2005; Pizzamiglio et al., 2005).

Because the associations between actions and sounds are important to human perception, behavioral studies have sought to uncover the nature of these associations. Castiello et al. (2010) showed that playing a priming sound before grasping an object sped up the execution of the grasping motion if the priming sound was the same as the sound produced by grasping the object. We recently performed a related series of experiments (Heller et al., 2012; Lemaitre et al., 2015), but with a paradigm that measured reaction time to cues that prompted different gestures. Participants were cued to initiate one of two gestures (e.g., tapping or scraping). Performing the gestures resulted in a response sound that was either naturally created (such as when a tapping gesture creates a tapping sound) or was artificially produced via an interface. Immediately before the gesture-instructing cue, a prime sound was played. The prime could be congruent, incongruent, or neutral with regard to the gesture. For example, a tapping sound being played before a tap cue would be congruent, while a scraping sound being played before a tap cue would be incongruent. Relative reaction times were significantly faster for congruent trials than incongruent trials, indicating that gestures can be primed by associated sounds.

Action-sound relationships have been examined to some extent, but little is known about the spatial frame of reference in which this particular association is made. In general terms, perceptual representation of spatial location can have an egocentric or an exocentric frame of reference (Klatzky, 1998). To describe the location of objects in space, an *egocentric* reference frame describes an object’s location with respect to the perceiver’s perspective. Conversely, an *exocentric* reference frame describes an object’s location independently of the perceiver’s perspective or location. For example, referring to a fellow automobile driver as being on your left side uses an egocentric frame of reference. However, referring to the location of the driver relative to the surface of the road would be an exocentric reference frame. Applying this distinction to action-sound associations, the frame of reference could in principle be *egocentric* by representing the action relative to the observers’ body, or it could be *exocentric* by representing the action relative to the environment, the external sound itself, or the artifact being used. For our purposes, action-sound associations that are represented *egocentrically* will be viewed in terms of self-generated gestures and thus integrated into the person’s body schema (Holmes and Spence, 2004), whereas action-sound associations that are represented *exocentrically* will be viewed in terms of motions applied to an object that produce a sound, represented relative to any external point of reference. Basic research into this distinction will help reveal a fundamental property of the psychological representation of actions and sounds. Additionally, the answer to this question could help guide the design of interfaces that utilize action-sound associations, as illustrated in the following examples.

Much of today’s technology makes use of the relationship between sound and gesture. When people press a button, swipe a screen, or plug in a device, they expect to hear something in response. If the response sound deviates from expectations (by perhaps being an “error”-type sound), users can tell that something has gone wrong. Likewise, if no sound is presented, individuals may question if the action was successfully performed. For example, delays in auditory feedback have been shown to impair the performance of musicians (Finney, 1997) as well as impair natural, complex movements, such running (Kennel et al., 2015). This important link between sound and gesture has been utilized by the technology industry to create user-friendly products, and it has been studied by researchers in multiple fields. For example, Caramiaux et al. (2014) showed that gestural descriptions of sound sources were more likely to involve actions (such as a crumple gesture) when the sound source was easy to identify (such as the crumpling of a piece of paper); such insights could lead to improved gestures in wearable computing if the gestures are matched with clearly identifiable sounds. Although distinguishing egocentric and exocentric viewpoints is important in usability, it has not yet been shown how they are manifested in action-sound associations.

The distinction between egocentric and exocentric is important for designing and understanding interfaces. Milgram and Kishino (1994) proposed a three-dimensional hierarchy of mixed reality virtual displays, in which one of the dimensions is Extent of Presence Metaphor, or simply how immersive the environment feels. This dimension directly corresponds to whether the virtual display is egocentric or exocentric, with the egocentric displays being more immersive, whereas more traditional interfaces such as the monitor-based “windows on the world” displays are completely exocentric and less immersive. Salzman et al. (1999) found that an egocentric frame of reference is beneficial for learning local, immersive details, but exocentric perspectives are better for more abstract, global concepts. Thus, they argue that a bicentric experience, which allows for alternating between the two, is superior. Likewise, Ferland et al. (2009) performed a study in which participants were asked to navigate a robot through various obstacles using an egocentric or exocentric 3D interface. Although egocentric viewpoints are useful for navigation, the exocentric reference frames are helpful in understanding the overall structure of the environment, and thus, they found that having access to both perspectives was beneficial to the task.

Whether action-sound associations are ego or exocentric has many implications for technology. First, if an immersive augmented reality is desired, action-sound associations should only be included if they are egocentric in nature, as exocentricity may make the experience feel less immersive (Milgram and Kishino, 1994). Additionally, if associations are egocentric, teaching action-sound associations should be done egocentrically (such as “use your thumb to play an F note on the clarinet” vs. “press the F key on the back of the clarinet”). As smart phones are now able to rotate their orientation, it is important to consider whether to design an interface egocentrically (relative to how a person is holding the phone) or exocentrically (relative to the phone). For example, swiping in an “up” gesture on a phone’s screen could raise the phone’s sound level. This is a simple association, but it is not immediately clear what should happen when the phone is rotated on its side or upside down. If action-sound associations are *egocentric*, then the phone should use its rotation sensor to account for the phone’s rotation and increase the sound level when swiped “up” *relative to how the user is holding the phone* (i.e., it might actually be to the left on the phone’s screen, for example). However, if action-sound associations are exocentric, then the interaction should be *relative to the phone’s screen*. Finally, gestural interfaces should be designed with the frame of reference in mind. Consider designing a musical device that generates pitches based on hand location. The hand location could be specified relative to the distance from the user’s body (egocentric) or relative to the distance from the floor (exocentric). If action-sound associations are egocentric, the first method would result in a more learnable and successful interface. Because the frame of reference is important for basic scientific understanding as well as for applications that utilize action-sound associations, we examined whether the action-sound relationships for computer keyboard users are egocentric or exocentric.

To address this question, a simple priming paradigm on a computer’s keypad was performed in which action-sound associations are created by pairing an action (keypress) with a sound (tone). For half of the participants, egocentric associations were introduced, and for the other half of participants, exocentric associations were introduced. All participants were executing the *same* action, and only the *framing* of the action varied, by altering the task instructions and directional cue. A priming paradigm was used to determine whether the association was learned in each condition. The egocentricity or exocentricity of action-sound associations was indicated by whether or not participants showed priming in each condition (i.e., if only the egocentric condition shows priming, we can conclude action-sound associations are egocentric in nature, and vice versa).

Part 2 further tests whether action-sound associations are egocentric or exocentric. The participants who showed an action-sound association halfway through the session (after part 1) were asked to switch to a different starting location (during the second half, part 2). If the association is purely egocentric, then changing to a different starting location will not change the results. Moving a finger “right,” for example, will be associated with the same sound, regardless of the finger’s starting location. *On the other hand*, if the associations are exocentric, moving to a new starting location will lower the effect size, as the new action-sound association would compete with the one that was just learned during part 1.

## Part 1

Part 1 of this experiment tests whether action-sound associations are created in egocentric or exocentric conditions. The frame of reference is varied by altering task instructions in half of the participants, and the strength of the associations is measured using a priming paradigm.

### Method

#### Participants

Participants were two groups of Carnegie Mellon University students recruited through an online psychology participant pool. Thirty-two English-speaking participants (17 female, 15 male) between the ages of 18 and 22 (median 19 years old) were in the egocentric experimental condition. Thirty-two participants (23 female, 9 male) between the ages of 18 and 21 (median 19 years old) were in the exocentric condition. The data from one 60-year-old participant were discarded in response to a reviewer’s request for our sample to match the customary age ranges used in RT experiments in the cognitive psychology literature; this removal did not affect the overall results.

All participants were right-handed with self-reported normal hearing and provided written informed consent prior to testing in accordance with procedures approved by the Carnegie Mellon University Institutional Review Board.

#### Interface and Apparatus

**FIGURE 1:**
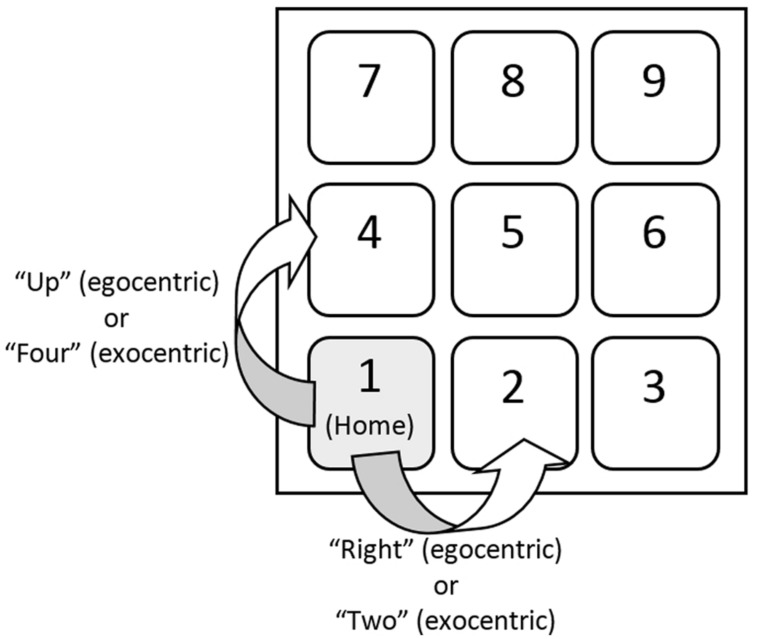
**General layout of the task in part 1.** Although moving one’s finger “right” and moving one’s finger to the “2” key are the same action, the frame of reference is differentiated as egocentric or exocentric by altering the directional cue. Likewise, moving one’s finger “up” or moving one’s finger to the “4” key are the same action but have different reference frames.

This experiment used an Apple USB keyboard (Model No: A1243), with tasks confined to the number keypad. **Figure 1** shows the general layout of the task. Digital sound files were converted to analog signals by an Audiofire 4 audio interface. All audio was presented over Sennheiser HD 600 open circumaural headphones.

#### Stimuli

Prime sounds consisted of a short low-pitched tone (534-Hz sinusoid) and high-pitched tone (1730-Hz sinusoid). Both sounds were enveloped using an Attack-Decay-Sustain-Release technique, with an attack time of 5 ms, decay time of 10 ms, sustain duration of 50 ms, and release time of 5 ms (total duration = 70 ms). The tones remained at a constant amplitude during the sustain portion. Sounds were presented at a 44100-Hz sample rate with 16-bit resolution.

Response sounds were identical to the prime sounds, with the low-pitched tone occurring when participants pressed the “2” key (via finger movement to the “right”), and the high-pitched tone occurring when participants pressed the “4” key (via finger movement in the “up” direction).

Directional cues were recorded via an Audio-Technica AT3525 30 Series microphone in an IAC double-walled sound-attenuating booth. They consisted of the vocal recording of an American English-speaking male saying the directions “right,” “up,” “two,” and “four.” The onsets of these directional cues were matched perceptually based on piloting, rather than by examining the waveform to account for differences in the slopes of the onset ramps (Tuller and Fowler, 1980). The onsets of the primes and responses sounds were perceptually and acoustically identical. All sounds were selected to have perceptually equal loudness.

#### Procedure

**FIGURE 2:**
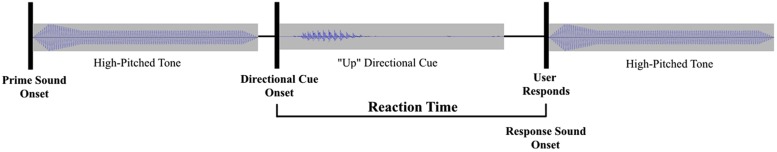
**Example trial structure.** The trial begins with a prime sound, then the directional cue is presented and the participant responds. In this example, the prime is congruent because the “up” gesture generates a high-pitched tone.

The structure of a trial is represented in **Figure 2.** Each trial started with the participants in the “home” position, which required holding down the “1” key on the number pad with their right index finger. After a short delay (400 ms), the prime sound was presented. This prime could be the high-pitched tone, the low-pitched tone, or a period of silence for the neutral condition. After a delay of 10 ms, the prime was followed by the vocal directional cue indicating which gesture to execute. The directional cue was “up” or “right” for the egocentric condition and “2” or “4” for the exocentric condition. When the participants responded, a response sound was played that always matched the gesture that was performed (but the response sound did not always match the prime sound). When participants moved their finger “up” (i.e., to the “4” key), the high-pitched tone was played, and when they moved their finger “right” (i.e., to the “2” key), the low-pitched tone was played. Participants were instructed to respond as rapidly as possible without sacrificing accuracy. Reaction times were measured from the onset of the directional cue.

It is important to note that the prime sounds were, by design, never predictive of which gesture would be required (while the response tone did always match the gesture). Half of the trials required an “up” gesture, while the other half required a “right” gesture. One-third of the trials used a congruent prime, one-third used an incongruent prime, and one-third had a neutral prime (silence that lasted the same duration as the tones). A congruent prime was one that matched the resulting response sound (for example, a high-pitched prime followed by an “up” response cue, as shown in **Figure 2**). Therefore, there were six types of trials (two response gestures × three prime-types). A total of 324 trials were presented to each participant in 18 blocks of 18 trials each. Each block was guaranteed to have three instances of each of the six trial types presented in different random orders for each block and participant. Following each trial, a recorded vocal message indicated whether the response was correct. Likewise, after each block, vocal recordings were provided to encourage faster reaction times, and visual feedback was displayed on the computer screen revealing the percent of correct answers and average reaction time.

Before beginning the main session, each participant watched a short, 12 trial demonstration of the experimenter performing the task. Next, participants familiarized themselves with the procedure in a preliminary training session of 72 trials (four blocks of 18 trials) in the presence of the experimenter. During this training session, the participants interacted with the experimenter to clarify the procedure. The experimenter ensured that the participants were executing the correct gestures and were responding correctly and as quickly as they could. The response sounds were audible during the training session, but the prime sounds did not begin until the main session.

### Results

Both accuracy and reaction time (RT) were measured. Raw RT data are available at https://zenodo.org/record/35563. A trial was considered incorrect if an incorrect key was pressed. RTs were measured from the onset of the directional cue and reflected the initiation of the movement away from the home position.

The preprocessing of RTs involved multiple steps. First, incorrect trials were removed. Next, outlier RTs were removed. The outlier cutoff was adjusted so that less than 0.5% of trials were excluded, based on the method described in Masson et al. (2011). The cutoff was established at 900 ms in the egocentric condition (0.491% of trials) and 990 ms in the exocentric condition (0.465% of trials). After preprocessing, each participant’s mean RT for each type of trial was calculated.

The neutral condition (silent prime) was used to correct for the inherent speed differences between the dominant and non-dominant hands. Silence was chosen because pilot attempts at finding a “neutral” cue sound failed to reveal a sound that was not biased toward one prime or the other. The choice to have a silent neutral condition prevents us from separating facilitation and inhibition effects, since the silence does not alert participants to the timing of the upcoming directional cue, and thus results in faster reaction times for primed trials compared to neutral trials. To account for the inherent differences between the gestures, we adjusted RTs by subtracting out the RTs for the neutral condition for each gesture and for each participant. This resulted in a measure of reaction time that was independent of gesture execution time, but this value was systematically negative (because neutral RTs were larger). Therefore, to appropriately characterize the relative reaction time between the two primed conditions, we added to this value the mean RT for the two primed conditions, averaged across all participants and conditions. The goal of this step was to produce positive numbers with the same average as the unprocessed RTs, which is easier to interpret than negative relative measures. The resulting *relative RT* is the average RT from trials with a prime *for a given gesture and a given prime* minus the RT for the baseline *for the same gesture* plus the average RT for any prime. This transformation allowed our analysis to be consistent with our previous research (Lemaitre et al., 2015). Note that, by definition, relative RT and raw RT produce the same statistics for the *congruency* variable (which was the main variable of interest). Relative RT does affect the *gesture* variable by subtracting out the baseline RT for each gesture, thus making the plots of congruency effects generalizable across a variety of gesture types (e.g., key presses, taps, and scrapes).

**FIGURE 3:**
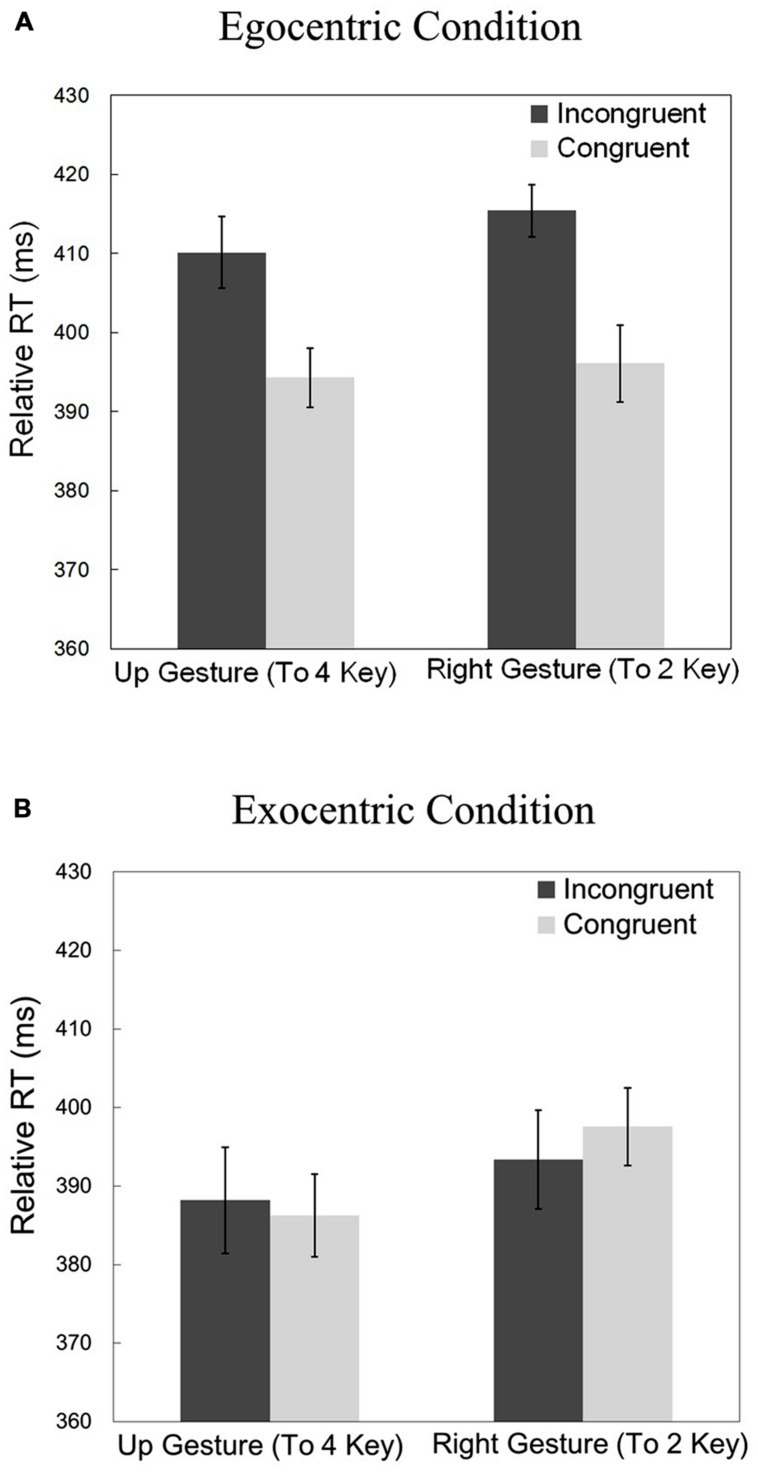
**(A)** Relative reaction times in part 1 for the egocentric reference frame condition (*gesture*-sound association). **(B)** Relative reaction times in part 1 for the exocentric reference frame (*key*-sound association). Vertical bars indicate the within-participant 95% confidence interval.

The relative RTs for the two gestures (“up” or “right”) and the two prime congruencies (congruent or incongruent) can be seen in **Figure 3.** Relative RTs were submitted to a repeated-measures ANOVA with the congruency and gesture as within-participant factors, the reference frame (egocentric or exocentric) as a between-participant factor, and the relative RTs as the dependent variable. There was a significant main effect of congruency [*F*(1,62) = 16.088, *p* < 0.01, η^2^ = 0.0655]. This shows that there were significantly longer relative RTs for incongruent cues versus congruent cues (i.e., priming was observed). There was a significant main effect of reference frame [*F*(1,62) = 12.334, *p* < 0.01]. Analysis also revealed that there was a significant interaction between congruency and reference frame [*F*(1,62) = 20.758, *p* < 0.01, η^2^ = 0.0845]. **Figure 3** illustrates that the effect of congruency is larger for the egocentric condition than the exocentric condition. There was also a significant main effect of gesture [*F*(1,62) = 4.828, *p* < 0.05, η^2^ = 0.0337]. There were no significant interactions between gesture and reference frame [*F*(1,62) = 0.768, *p* = 0.384], between gesture and congruency [*F*(1,62) = 0.219, *p* = 0.641], nor between gesture, congruency, and reference frame [*F*(1,62) = 2.906, *p* = 0.093].

Because of the significant interaction between congruency and reference frame, it is important to look at the two reference frames separately. An ANOVA was performed for just the egocentric condition, with gesture and congruency as between-subject factors and relative RT as the dependent variable. There was a main effect of congruency [*F*(1,31) = 43.772, *p* < 0.01, η^2^ = 0.3508]. There was no significant main effect of gesture [*F*(1,31) = 1.537, *p* = 0.224] and no significant interaction between congruency and gesture [*F*(1,31) = 1.135, *p* = 0.295].

Likewise, a similar ANOVA was completed for just the exocentric condition. Here there was *not* a significant main effect of congruency [*F*(1,31) = 0.128, *p* = 0.723]. There was also not a significant main effect of gesture [*F*(1,31) = 3.297, *p* = 0.079] nor was there an interaction between congruency and gesture [*F*(1,31) = 1.780, *p* = 0.192].

Overall, accuracy was high, with an average of 98.1% (*SD* = 2.1%), with a minimum of 95% across all conditions. The uniformly high accuracies suggest that a substantial speed–accuracy tradeoff is unlikely.

### Discussion

Because priming was observed in the egocentric condition, we can conclude that an egocentric association existed between the sounds (high-pitched and low-pitched tone) and the gestures (”right” and “up”). However, priming was not observed in the exocentric condition, providing no evidence for exocentric key-sound associations. This suggests that these action-sound associations are egocentric in nature.

## Part 2

To further test whether action-sound associations are egocentric or exocentric, part 2 explores how altering the starting location affects the strength of the associations. If the associations are egocentric, then changing to the new starting key will not affect the results. Moving a finger “right,” for example, will be associated with the same sound, regardless of the finger’s starting location. Conversely, if the associations are exocentric, moving to a new starting location will lower the effect size, as the new action-sound association would compete with the one that was just learned during part 1.

In order to see if the change in starting location lowered the effect size, it was necessary to only include participants who showed an individual priming effect in part 1. Each participant’s data was analyzed to examine if there was an individual priming effect. Because the egocentric (but not the exocentric) condition in part 1 showed an overall priming effect, it was expected that most participants who did show an individual priming effect would be within the egocentric condition.

### Method

#### Participants

After the first 324 trials (part 1), inclusion criteria for part 2 were applied. The Cohen’s *d* was determined for each participant for each gesture in order to judge the effect size (Cohen, 1988). This value was calculated by dividing the difference between incongruent and congruent relative RTs by the pooled standard deviation of the individual participants’ relative RTs in those conditions. Participants showing at least a small effect size (a Cohen’s *d* of at least 0.2 for both gestures) were moved onto part 2. Part 2 used the “5” key as the starting position, and participants moved their fingers “right” (to the “6” key now) or “up” (to the “8” key now). The participants that did *not* meet the inclusion criteria continued working on part 1 (data from which are not presented). Only including the participants that already had acquired a modest priming effect was vital to the design of the experiment. In order to appropriately compare the first starting position to the second starting position, an effect was necessary at the start.

Of the 32 participants in the *egocentric* condition from part 1, 11 met the inclusion criteria for part 2 at the end of their 324 trials. These 11 participants (eight female, three male) were between the ages of 18 and 22 (median 19 years old). Of the 32 participants in the exocentric condition from part 1, only two participants had a Cohen’s *d* of at least 0.2 for both gestures (one of whom was not switched to the new starting position in part 2, due to error). The very small number of participants in the exocentric condition who passed the inclusion criteria was consistent with the lack of priming effect observed in part 1 and supports the interpretation of this null effect as being a result of the absence of a priming effect in the population rather than being due to variability of a priming effect in the population.

#### Interface and Apparatus

**FIGURE 4:**
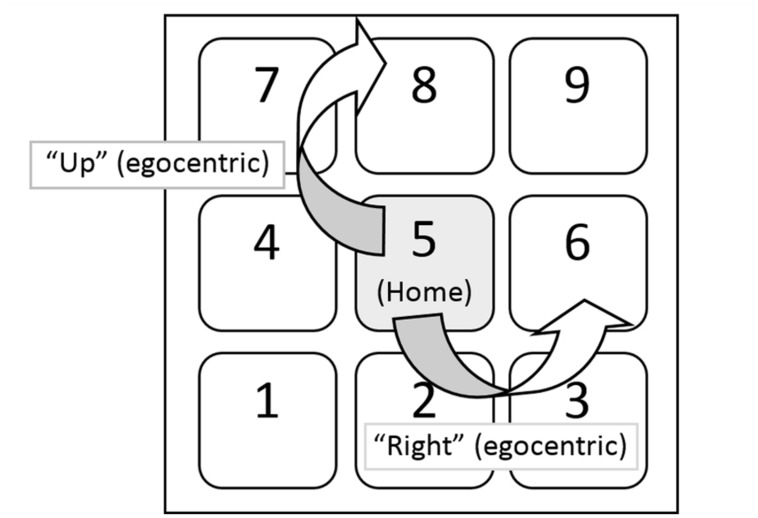
**General layout of the task in part 2.** Now instead of starting at the “1” key, participants were required to start at the “5” key. Note that if the association is egocentric, then there will be no effect of starting location.

Part 2 of this experiment used the same interface as part 1, with the exception that participants now started on the “5” key. Participants were still instructed to move their finger “right” or “up” for the egocentric condition. **Figure 4** shows the layout of this part of the experiment.

#### Stimuli

Prime and response sounds were the same low-pitched and high-pitched tones from part 1. Response sounds were mapped to the same gestures as in part 1 for the egocentric condition: The low-pitched tone occurred when participants moved their finger “right,” and the high-pitched tone occurred when they moved their finger “up.” The gestures were identical for both starting positions, and only the keys differed. Directional cues were the same “right” and “up” recordings from part 1 for the egocentric condition. The single participant from the exocentric condition who continued on to part 2 received exocentric cues which were vocal recordings from the same English speaker saying “6” and “8”.

#### Procedure

Participants began part 2 after a short break. The procedure of part 2 of the experiment was identical to part 1, except participants began trials with the “5” key held down. Participants were still instructed to either move their finger “right” (i.e., to the “6” key) or “up” (i.e., to the “8” key). Again, there were 324 trials, although no practice was given (only verbal instructions).

## Results

The 11 participants from the *egocentric* condition in part 1 who met the inclusion criteria contributed data from both parts 1 and 2 to the subsequent analysis. Because data were available from only one participant in the *exocentric* condition, we do not present an analysis of the effect of changing starting position in part 2 of the exocentric condition, but we note that the individual effect size from that one participant decreased in part 2.

**FIGURE 5:**
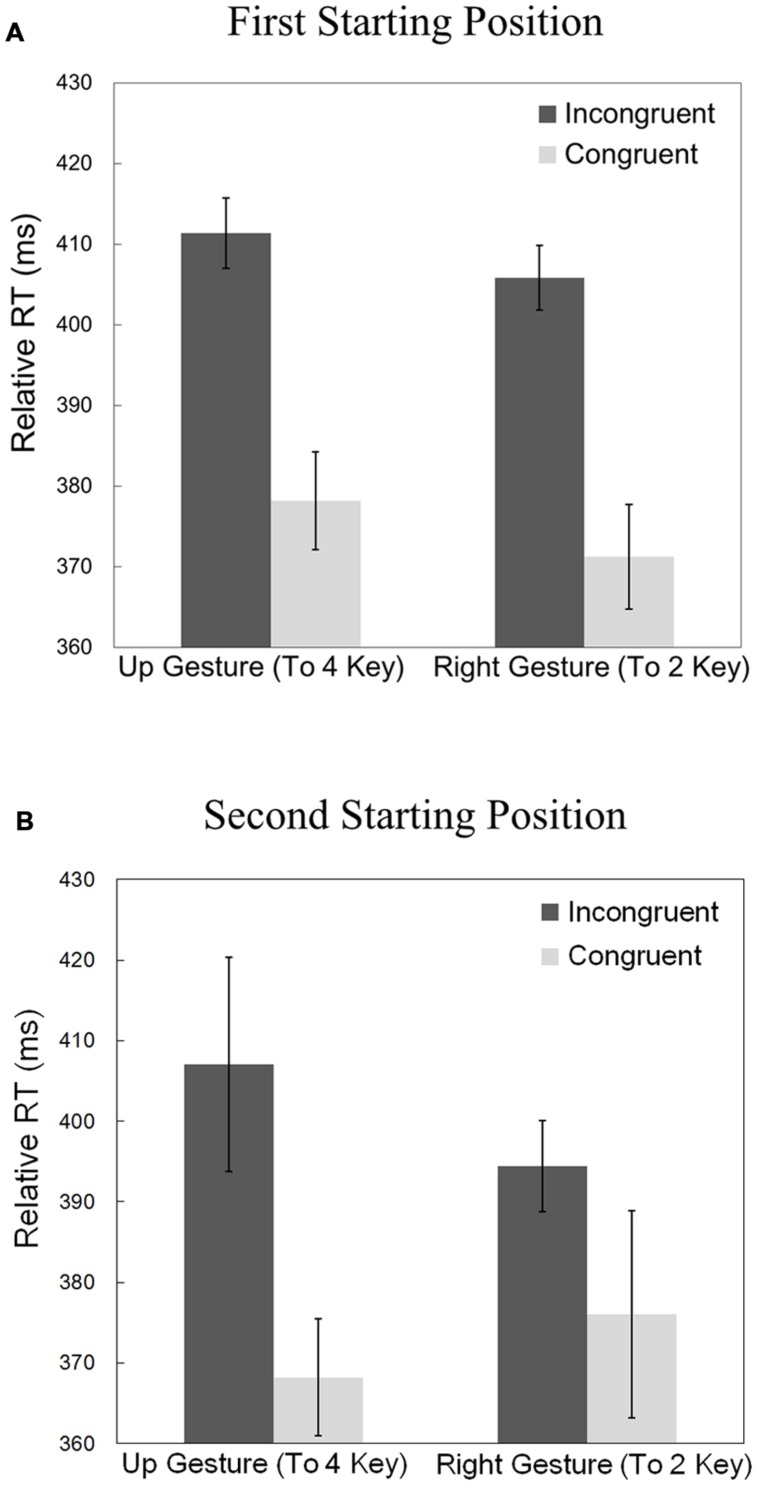
**(A)** Relative reaction times in part 1 (first starting position), including only the participants who had a Cohen’s *d* of at least 0.2 for both gestures (*n* = 11). **(B)** Relative reaction times for part 2 (second starting position), using same participants as **(A)**. Vertical bars indicate the within-participant 95% confidence interval.

The preprocessing of the RT data was identical to that of part 1. **Figure 5** displays the relative RTs for the two gestures (“right” and “up”) and the two prime congruencies (congruent or incongruent) for the two starting positions. Relative RTs were submitted to a repeated-measures ANOVA with the congruency, gesture, and the starting point (“1” key or “5” key) as within-participant factors and the relative RTs as the dependent variable. There was a significant main effect of congruency [*F*(1,10) = 62.56, *p* < 0.01, η^2^ = 0.4299]. There was *not* a significant main effect of starting point [*F*(1,10) = 0.565, *p* = 0.470]. There was also no significant interaction between congruency and starting point [*F*(1,10) = 0.811, *p* = 0.389], indicating that the way in which congruency affected relative RTs did not depend on starting point. In other words, the priming effect was not significantly different between the two starting locations. There was not a significant main effect of gesture [*F*(1,10) = 0.912, *p* = 0.362], interaction between gesture and congruency [*F*(1,10) = 3.390, *p* = 0.095], nor interaction between gesture and starting location [*F*(1,10) = 0.222, *p* = 0.648]. There was a difficult-to-interpret three-way interaction between congruency, gesture, and starting location [*F*(1,10) = 12.668, *p* < 0.05, η^2^ = 0.0132].

As was found in part 1, the overall accuracies for part 2 were high, with an average of 97.6% (*SD* = 2.5%), with a minimum of 95% across all conditions. The uniformly high accuracies suggest that a substantial speed–accuracy tradeoff is unlikely.

### Discussion

The absence of a significant main effect of starting location and the lack of a two-way interaction between congruency and starting location suggest that the priming effect did not depend on starting position. These results are consistent with the conclusion from part 1 which suggests that the created action-sound associations are egocentric. Egocentric gestures are represented the same way regardless of starting location because they are relative to the agent.

## General Discussion

Part 1 of this study asked whether action-sound associations are created in egocentric or exocentric conditions. A priming effect relating keypresses to sounds was observed in the egocentric condition, which demonstrates that an association between a gesture and a sound can be created. However, no priming effect was observed in the exocentric condition, which suggests that these action-sound associations are egocentric in nature. Furthermore, part 2 demonstrated that there is no observable difference in association strength when the starting position of an egocentric gesture-sound association is changed, which is predicted by an egocentric reference frame. Taken together, the results of this experiment support the idea that action-sound associations, specifically those of keypresses and tones, are egocentric, rather than exocentric.

It is worth noting that it is conceivable that the effect size should have been even *larger* after moving the starting position for the egocentric condition, simply because participants would have had more previous trials to learn the association, and thus, have had a stronger association in the second half of the experiment. However, it is likely that the participants had reached a plateau in their learning curve (Thurstone, 1930), as these are simple gesture-sound associations and the effect sizes at the end of part 1 were similar in magnitude to the effect sizes observed in our previous experiments (Heller et al., 2012; Lemaitre et al., 2015). Nonetheless, further experiments are necessary to fully characterize the learning curve of these associations.

It may be initially surprising that only 11 of the 32 participants in the egocentric condition in part 1 of this study showed a Cohen’s *d* of at least 0.2 for both gestures, despite the significant priming effect at a group level. The group statistics typically used in priming experiments, such as ANOVA, do not depend upon individuals showing reliable effects. Most psychology studies do not even report the percentage of participants who show an individual effect, so it is difficult to compare our results to similar experiments. One study tested three inhibition/interference tasks (Stroop color, Negative priming embedded within the Stroop color, and Hayling) and found significant effects at a group level, but quite variable percentages of individuals who showed an effect (Borella et al., 2009). The percentages were determined using a bootstrap method and were as low as only 17% of individual participants showing an effect, despite a significant group effect, which is comparable to the 34% in our study.

It may also be surprising that the participants in the exocentric condition in part 2 did not remap their task instructions into egocentric terms, which would have resulted in a priming effect. One explanation is the fact that the directional cue, which was played on every trial, was either “2” or “4.” This continuously emphasized the exocentric reference frame. Post-test questioning of the participants in the exocentric condition revealed that they associated a *key* with a tone, even for the two participants who met our criterion effect size, which indicates that those individuals were not explicitly remapping the association to a gesture.

A limitation of our current study is that we are unable to separate the facilitation and inhibition components of the priming effect. Future experiments could include a neutral prime condition with a sound that is carefully designed so as to have no perceived similarity to any of the priming sounds or gestures.

An alternative explanation for our results could be that the prime sound may be priming an *abstract representation* of the gesture (or decision to move) and not necessarily *the gesture itself*. A similar idea was proposed by de Wit and Kinoshita (2014) to explain their study in which relatedness proportion affected the size of a semantic priming effect, which could not simply be explained by an automatic spreading of activation. They argue for an explanation that is based on an evidence accumulation process and source confusion between the prime and target. The decision to move to a target is facilitated when the evidence from the prime is congruent with that needed for the decision, which could indeed be an explanation for our results. However, the limitations of our study prevent us from disentangling these possible explanations. Furthermore, Elsner and Hommel (2001) used a free-choice response task (i.e., with no imperative cue) to show that sounds associated with an action can prime action even when there is no cue mediating the decision. Regardless of the interpretation, the important fact remains that priming was only observed for a gesture-based movement and *not* a key-based movement.

Because the associations in our study paired a high-pitch with the “up” gesture and a low-pitch with the “right” gesture, it is important to consider the possibility that a SNARC or SMARC effect is being observed, in which the association is caused by an implicit pairing of the tones to the specific response gestures. The SNARC/SMARC effect (Rusconi et al., 2006) reveals a cognitive favoring of high pitches with responses on the right side of a horizontal plane (and toward the top of a vertical plane) and a favoring of low pitches with responses on the left side of a horizontal plane (and toward the bottom of a vertical plane). In the Rusconi et al. (2006) study, participants were faster to respond to congruent trials (e.g., a high pitch requiring a response on an upper key) compared to incongruent trials (e.g., a high pitch requiring a response on a lower key). This was true even when the pitch was not relevant to the task. However, this effect is not likely to be relevant to the results of our study. The SNARC/SMARC effect states that high pitches are associated with up *and* right, while low-pitches are associated with left *and* down. Because we used up and right gestures, those would *both* be associated with higher pitches, resulting in no preference for either pitch. Additionally, while our study did not counterbalance the pairing between tones and gestures, previous experiments from our lab did counterbalance the same pitches and gestures, finding no effect of pairing, which suggests that an implicit association is unlikely (Lemaitre et al., 2015).

Although little is known about the connection between auditory action perception and frame of reference, one recent study has found a relationship between sounds and egocentricity. Tajadura-Jiménez et al. (2015) has shown that auditory feedback (i.e., the auditory distance of action sounds) can manipulate the mental representation of the self (i.e., arm length). There is a possibility that all cognition involved with auditory action perception is inherently egocentric, but significant research is needed to start exploring this question.

Egocentric action-sound associations have many potential psychological applications. Altavilla et al. (2013), for example, have recently devised an application that utilizes associations between sounds and egocentric actions. They have shown that users are able to learn that doing certain gestures while wearing a sensor creates different types of sounds. Users are then able to recreate heard sounds by performing the gestures that would cause them. In other words, participants learned the associations between doing a gesture and creating a sound. Importantly, the gestures used in their study were egocentric (e.g., a vertical tilt of the hand). It could be postulated that the success of their study is due to the fact that the gestures were egocentric, and that if exocentric actions were used instead, users may have difficulty learning the action-sound associations.

Likewise, Serafin et al. (2014) created a mapping strategy between sensor-equipped gloves and sound synthesis models. Their approach was based on embodied music cognition, which focuses on the role of the human body in relation to musical activities. Based on our findings, this approach is justified. Action-sound associations that are based on egocentric actions appear more learnable than ones based on exocentric actions.

Finally, Caramiaux et al. (2014) showed that the gestural description of a sound stimulus depends on the identifiability of the causal action of the sound source. They found that participants mainly mimic the action that produced the sound when the action is known. However, when the cause of the sound was unknown, participants traced contours related to sound acoustics. Their finding could lead to applications that use acoustic contours in sound synthesis. Based on our findings, we suggest that these contours be egocentric. That is, they should be relative to a point on the user (such as bellybutton), and not relative to some external object (such as a countertop).

We have found evidence favoring the formation of egocentric action-sound associations (i.e., a body-centric gesture producing a tone) over exocentric ones (i.e., a specific key depression producing a tone). This suggests that the action-sound associations are fundamentally represented in terms of the gestures that produce the sounds. Additionally, changing the starting location of previously learned gesture-sound associations does not change the strength of the association, which further suggests that action-sound associations are egocentric in nature. As technology begins to pair gestures and sounds more and more, the advantages of understanding the egocentricity of these associations can be realized.

## Author Contributions

NN contributed the idea, helped design the experiment, recruited and ran participants, performed data analysis, and prepared manuscript and figures. GL helped with data analysis and helped review manuscript. AF contributed to initial discussions on the topic and helped review manuscript. LH contributed to all phases of the experiment.

## Conflict of Interest Statement

The authors declare that the research was conducted in the absence of any commercial or financial relationships that could be construed as a potential conflict of interest.
